# Detection of urban hidden faults using group-velocity ambient noise tomography beneath Zhenjiang area, China

**DOI:** 10.1038/s41598-020-80249-6

**Published:** 2021-01-13

**Authors:** Leiming Zheng, Xiaoping Fan, Peng Zhang, Jingrun Hao, Hao Qian, Tuo Zheng

**Affiliations:** Nanjing Tech University, Nanjing, 211816 Jiangsu China

**Keywords:** Solid Earth sciences, Engineering

## Abstract

The Mufushan-Jiaoshan fault (MJF) is a hidden active fault located on the north side of the Ningzhen Mountain Range and developed along the Yangtze River in Zhenjiang area, China. In this paper, the structure of MJF is detected and studied using group-velocity ambient noise tomography. In the study area (18 km × 25 km), 47 short-period seismic stations were deployed with the average station spacing of about 3 km and 24 days (from 27 February to 22 March 2019) of continuous ambient-noise recordings were collected. And 510 group velocity dispersion curves in the period band 0.5–5 s were extracted using the vertical component data. And then the three-dimensional shear-wave velocity structure was inverted using group dispersion data by the direct surface-wave tomographic method. Our results are consistent with the geological background of the study area, showing that in the depth range of 0.6–1.5 km, the north side of MJF presents a relatively high velocity, and the south side presents a distribution pattern of high and low velocity. While in the depth range of 1.5–2.0 km, the shear-wave velocity (V_s_) model is relatively simple with relatively low velocity on the north side and relatively high velocity on the south side. And the gradient zone of V_s_ may be the location of the main fracture surface of MJF. The good correspondence between the V_s_ model and the fault structure indicates that the ambient noise tomography method can be used as an effective method for detecting hidden faults in urban environments.

## Introduction

The detection of urban hidden faults is an essential work for city planning, construction and earthquake disaster reduction especially when urbanization is highly developed and any strong earthquake (e.g. the 2008 magnitude 7.9 Wenchuan earthquake and the 2010 magnitude 7.0 Haiti earthquake) happened in urban areas will cause immeasurable losses^1–3^. To study the structure of hidden faults, there are generally two methods, comprehensive active source experiments and passive seismic monitoring. However, due to the dense population and complex environment in urban area, many problems arise, such as source selection, the layout of survey lines, and the interference problem, thus it is difficult to get reasonable results using the traditional comprehensive active source experiments. And because of the scarcity of earthquakes, the high solution result can neither be obtained using the passive seismic monitoring.

However, using ambient noise tomography (ANT) with a reasonable array design, we can also get the high-resolution subsurface structure in a certain depth range without conducting active source experiments and may obtain the structure of the hidden faults before earthquakes occur. The basic idea of ANT is to obtain the time cross-correlation functions (CFs) or the time domain empirical Green’s functions (TDEGFs) between each station pair, which contain information about the structure of the crust and upper mantle between two stations^4–6^. And the CFs or TDEGFs can be used to retrieve the surface waves between stations, which can then be used to invert high-resolution velocity structure at different depths^7,8^. Since its first implemented in southern California in 2005, ANT has been widely used all over the world and surface wave data of 5–40 s is generally used to invert crust and upper mantle structure^7–12^. For the last few years, however, many studies begin to pay attention to invert shallow crustal or near-surface structures using shorter period surface wave data, mostly in the period band 0.2–5 s^13–21^.

In this study, in order to detect the structure of the Mufushan-Jiaoshan fault (MJF), which is located in the urban area of Zhenjiang, China, and the location of its main fracture surface, 47 stations were installed across the Yangtze River and continuous ambient-noise data for 24 days (from 27 February to 22 March 2019) were collected.

## Tectonic setting

**Figure 1:**
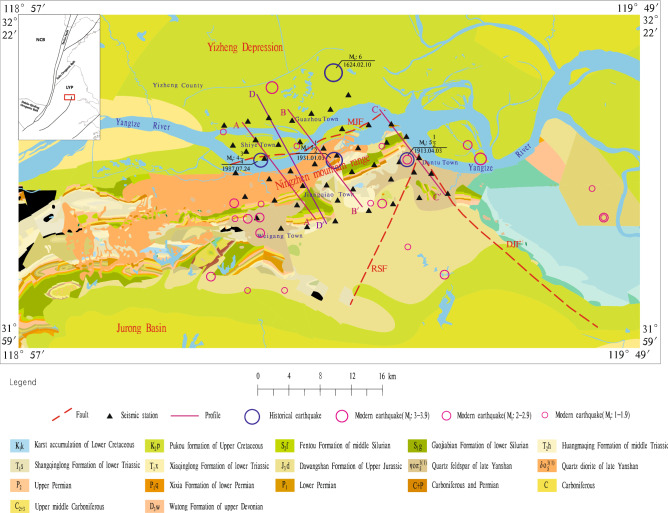
Geologic structure, faults, river systems, earthquake location, and station location in the study area and its adjacent regions. The inset figure shows the locations of the study area and its adjacent regions (red box) and the NCB, LYP represent North China Block, and Lower Yangtze Plate, respectively. (The software used to create the map is MAPGIS 6.7, http://www.mapgis.com.cn/).

The Zhenjiang area is part of the east section of the Ningzhen Mountain Range (NMR) and is located in the Yangtze River Delta region of China, which is one of the most economically vibrant and urbanized areas in China. The Zhenjiang area has a complex terrain, with the new Yangtze River Delta plain in its northern and eastern parts, low mountains and hills in its central part, and landform in its southern part (Fig. 1).

Since the Indo-Chinese movement, the Zhenjiang area has undergone multi-period and multi-stage structural changes, which leading to the development of several large-scale fault structures, including the Mufushan-Jiaoshan fault (MJF), the Rushan-Shanghui fault (RSF), and the Dantu-Jianshan fault (DJF)^22^.

The NMR is the main geological structure in the study area, controlling the formation and development of the main fault structure in the area. In the early Mesozoic, the North China plate collided with the Yangtze plate, forming the NMR, and generating a series of northeast trending, nearly east–west trending nappe structures and fold uplifts; from the late Jurassic to the early Cretaceous, the Pacific plate subducted to the west, and strong faulting occurred in the area, which caused a wide range of magma effusion and intrusion^23^; after the Late Cretaceous, due to changes in the tectonic stress field, northeast-northeast to east and northwest faults, such as the MJF, the DJF, and the RSF, were further formed based on the original N-E and near E-W arc structures.

The MJF generally strikes in the E-W direction, with the fracture surface inclined to the north. The south side of the fault is NMR and the north side is Yizheng depression. Due to the long-term activity of the MJF, large-scale depressions occurred in the northern half of the anticlinoriums, such as Mufu mountain and Qixia mountain, forming the Yizheng depression and the Ningzhen fault block uplift, which have undergone significant block differential uplift movement along the MJF^22^.

These fault structures not only control the Quaternary sedimentary environment, but also are related to the multiple destructive earthquakes. For example, the MJF, the RSF, and the DJF intersecting near Jiaoshan are believed to be related with the 1624 magnitude 6.0 Yangzhou earthquake, the 1913 magnitude 5.5 Zhenjiang earthquake, and the 1930 magnitude 5.0 Zhenjiang earthquake^22,24^.

## Ambient noise data analysis

**Figure 2:**
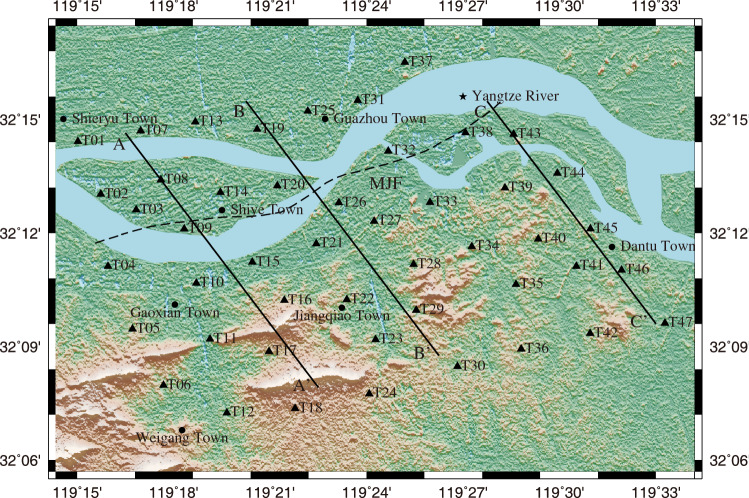
Distribution of seismic array stations (black triangles) in Zhenjiang area. The black lines marked by AA’, BB’ and CC’ represent the profiles shown in Figs. 9 and 10. The dotted black line represents MJF. (The software used to create the map is GMT V5.4.5, https://www.generic-mapping-tools.org/).

From 27 February to 22 March 2019 (24 days), 47 short-period seismic stations, with the average spacing of about 3 km, were deployed across the Yangtze River in the urban area of Zhenjiang area (about 18 km × 25 km). The distribution of seismic array stations and the topography of the study area are shown in Fig. 2. And then the vertical component data of the 24-day continuous ambient-noise recordings were selected to invert the Vs model of the study area by ANT.

**Figure 3:**
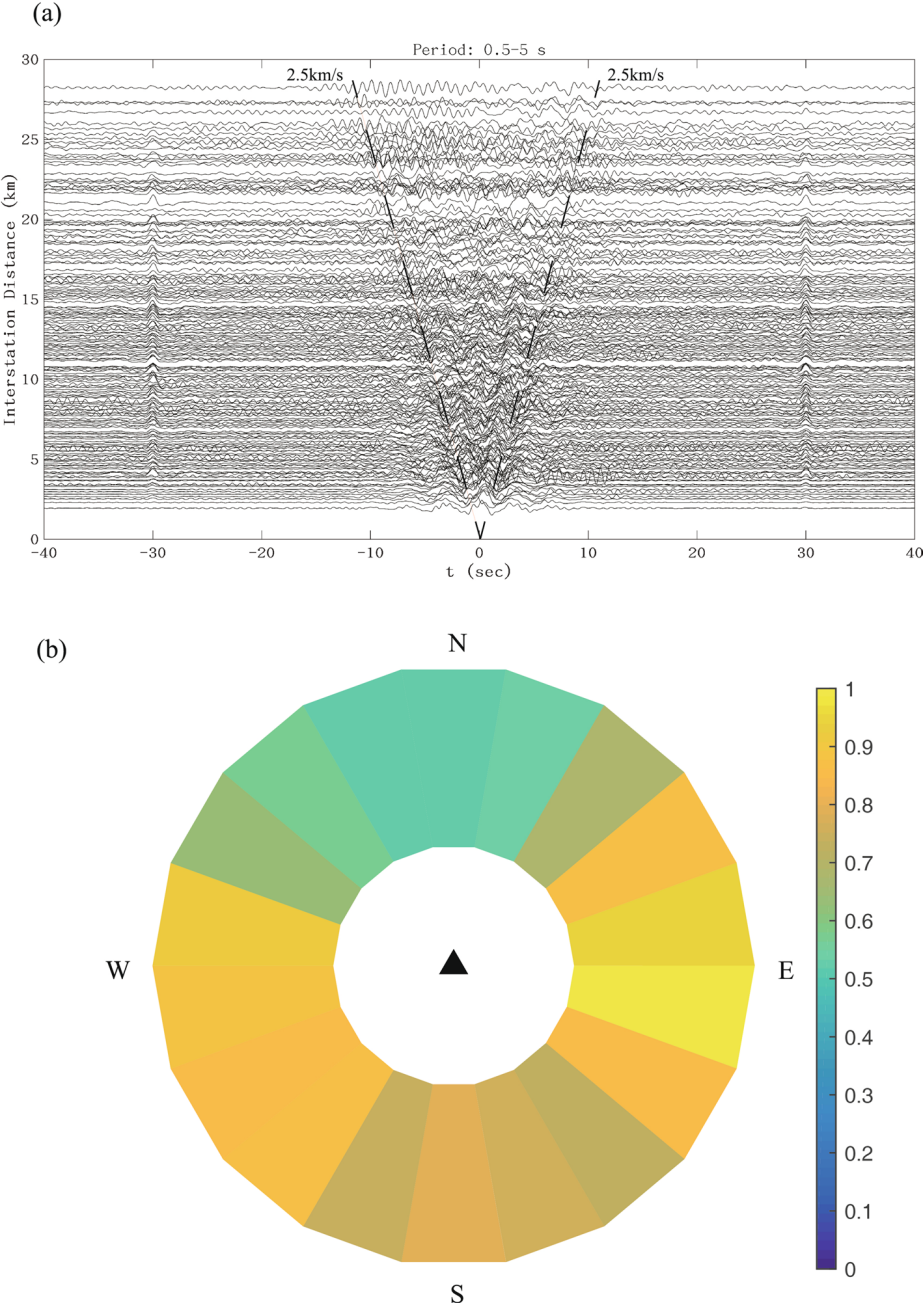
(**a**) Cross-correlation functions (CFs) with signal-to-noise ratio (SNR) greater than five in the 0.5–5 s period band obtained from normalized linear stacking method. (**b**) Azimuthal dependence of the normalized amplitudes of CFs for all station pairs in the 0.5–5 s period band.

In order to get more reasonable results, the standard ambient noise processing procedure^25^ were followed to process our data. Firstly, we re-sampled the vertical component data, which was cut into hourly segments, at a sampling rate of 10 Hz to reduce the cost of computational time. Secondly, we removed the mean and trend of the data, and also band-pass filtered the data in the frequency band from 0.5 to 5 s. Thirdly, after these steps, to suppress the effects of earthquakes, the spectral whitening and temporal normalization were performed^26^. Finally, we conducted cross correlation to obtain the time domain cross-correlation functions (CFs) between two stations for each hourly data with the lag time from − 40 to 40 s. Using normalized linear stacking method, we stacked all the hourly CFs from each station pair and obtained the time-domain empirical Green’s functions from the time derivative of the CFs^8,9^. Figure 3a show the CFs with signal-to-noise ratio (SNR) greater than five in the period band 0.5–5 s after stacking and the surface-wave signals of about 2.5 km/s can be clearly observed. Here, the SNR is defined as the ratio of the maximum amplitude of the signal window of CFs (− 20 to ~ 20 s) and the average absolute amplitude of the noise window (− 40 to ~ 20 s and 20 to ~ 40 s). In addition, the additional arrivals with higher velocities (5 km/s) might be higher-mode surface waves and the phases at − 30 s and 30 s might be related to some kinds of interference signals, which cause little impacts on the cross-correlation calculation results^27^. In the period band of 0.5–5 s used in this study, the ambient noise is usually regarded to be related to the man-made sources (e.g., traffic, cultural, and human activities), the scattering from teleseismic earthquakes, and the “leaking” from some second microseismic peak^28,29^. To check the distribution of the ambient noise sources in the study area, the normalized amplitude of the positive- and negative- time CFs for all station pairs were computed (Fig. 3b). The ambient noise sources from the east are slightly stronger than those from other directions (Fig. 3b), which may be related to the East China Sea. But from the overall perspective, most values of normalized amplitudes are between 0.5 to 1.0, which means that there are small amplitude differences between positive- and negative-time CFs and that the noise source distribution will not cause large bias in dispersion measurements^30^.

**Figure 4:**
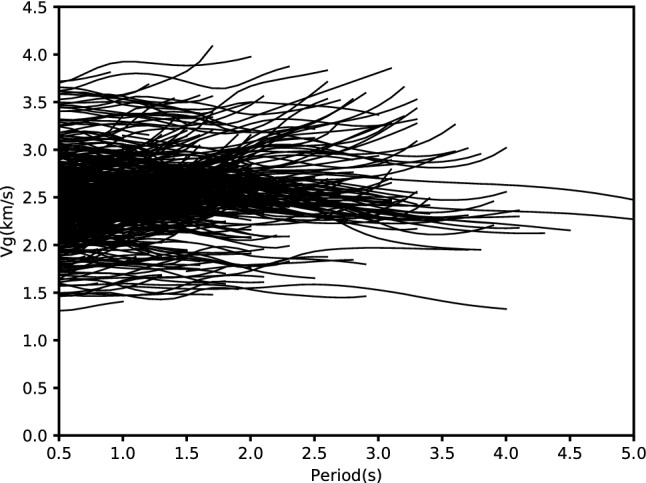
Group velocity dispersion curves in the 0.5–5 s period band.

After stacking the positive-time and negative-time parts of the time-domain empirical Green’s functions linearly, we used the quick tracing method EGFAnalysisTimeFreq based on frequency-time analysis and image analysis technique to first construct the velocity-period spectrogram for each TDEGF and then extract the fundamental mode Rayleigh wave group velocity dispersion curves of each station pair by picking the peak of the envelope function of the narrow-band filtered signal^31,32^. In order to satisfy the far field approximation of surface wave propagation, the interstation distance is required at least 2 times the wavelength^31^. All the 510 group velocity dispersion curves in the period band 0.5–5 s for the fundamental mode Rayleigh waves are shown in Fig. 4. It can be seen that, the quantity of dispersion data decreases with increasing period and there is no measurement can be made above the 5 s period due to relatively short interstation distance. In addition, as inferred from the dispersion curve, the velocity variation in the study region appear to be very large; For example, at the period of 0.5 s, group velocity varies from 1.3 to 3.7 km/s.

## Method

In this study, we used the direct surface-wave tomography method, which is based on frequency-dependent ray tracing and a wavelet-based sparsity- constrained tomography inversion, to invert group velocity dispersion data for the 3D shear velocity structure^33^. In comparison with the traditional two-step surface wave inversion method, this method avoids the intermediate step of inversion for group velocity maps and considers ray-bending effects of surface waves due to complex velocity structure based on the fast-marching method^34^.

The goal of this method is to find a model **m** that minimizes the differences $\updelta {t}_{i}\left(\omega \right)$ between the observed times ${t}_{i}^{obs}\left(\omega \right)$ and the model prediction ${t}_{i}\left(\omega \right)$ for all frequencies $\omega$ and the travel-time difference at angular frequency $\omega$ with respect to a reference model for path **i** is given by Fang, et al.^33^

1 $$ {\updelta }t_{i} \left( \omega \right) = t_{i}^{obs} - t_{i} \left( \omega \right) \approx - \mathop \sum \limits_{k = 1}^{K} v_{ik} \frac{{\delta C_{k} \left( \omega \right)}}{{C_{k}^{2} \left( \omega \right)}}, $$

where ${t}_{i}(\omega )$ is the calculated travel time from a reference model which can be updated in the inversion, and ${v}_{ik}$ are the bilinear interpolation coefficients along the ray path associated with the **i**th travel-time data, the group velocity ${C}_{k}\left(\omega \right)$ and its perturbation $\updelta {C}_{k}(\omega )$ at the **k**th two-dimensional (2D) surface grid point at angular frequency $\upomega$. The group velocity perturbation can be written as

2 $$ {\updelta }C_{k} \left( \omega \right) = \int {\left[ {\frac{{\partial C_{k} \left( \omega \right)}}{{\partial \alpha_{k} \left( z \right)}}\left| {{}_{{\theta_{k} }}^{{}} \delta \alpha_{k} \left( z \right) + \frac{{\partial C_{k} \left( \omega \right)}}{{\partial \beta_{k} \left( z \right)}}\left| {{}_{{\theta_{k} }}^{{}} \delta \beta_{k} \left( z \right) + \frac{{\partial C_{k} \left( \omega \right)}}{{\partial \rho_{k} \left( z \right)}}\left| {{}_{{\theta_{k} }}^{{}} \delta \rho_{k} \left( z \right)} \right.} \right.} \right.} \right]} d_{z} $$

Based on the empirical relationships proposed by Brocher^35^, compressional wave velocity and density are related to shear wave velocity, thus Eq. (1) can then be expressed as

$$ \delta t_{i} \left( \omega \right) = \mathop \sum \limits_{k = 1}^{K} - \frac{{v_{ik} }}{{C_{k}^{2} \left( \omega \right)}} $$

$$ \mathop \sum \limits_{j = 1}^{J} \left[ {\left. {R^{\prime}_{ \propto } \left( {z_{j} } \right)\frac{{\partial C_{k} \left( \omega \right)}}{{\partial \propto_{k} \left( {z_{j} } \right)}} + R^{\prime}_{\rho } \left( {z_{j} } \right)\frac{{\partial C_{k} \left( \omega \right)}}{{\partial \rho_{k} \left( {z_{j} } \right)}} + \frac{{\partial C_{k} \left( \omega \right)}}{{\partial \beta_{k} \left( {z_{j} } \right)}}} \right|} \right]_{{\theta_{k} }} $$

3 $$ {\updelta }\beta_{k} \left( {z_{j} } \right) = \mathop \sum \limits_{l = 1}^{M} G_{il} m_{l} , $$

where ${\theta }_{k}$ represents the 1D reference model at the **k**th surface grid point on the surface and ${\propto }_{k}\left({z}_{j}\right),$ ${\beta }_{k}\left({z}_{j}\right),$ and ${\rho }_{k}\left({z}_{j}\right)$ are, respectively, the compressional velocity, shear velocity, and mass density at the *j*th node in the depth direction, and $M=KJ$ represents the total number of grid points of the 3D model^33^. And ${R}_{\propto }^{^{\prime}}\left({z}_{j}\right)$ and ${R}_{\rho }^{^{\prime}}\left({z}_{j}\right)$ are the scaling factors derived from the empirical relationships^35^.

The direct surface-wave tomography Eq. (3) can be further expressed in matrix form as

4 $$ {\mathbf{d}} = {\mathbf{GM}}, $$

where $\mathbf{d}$ represents the surface-wave travel-time residual vector for all ray paths and periods, and $\mathbf{G}$ and **M** represent the data sensitivity matrix and the model parameter vector, respectively.

## Inversion details and model resolution tests

We here used the group dispersion data and an average 1D model, which was determined using the average group velocity dispersion curves, as the initial model to invert the near surface shear velocity structures. For the inversion grid, we parameterized the study area into 18 by 18 grid points on the horizontal plane and 9 grid points along the depth direction. The grid intervals along the latitude and longitude are 0.014° and 0.025°, respectively. At the depth direction, 9 grid points were set from 600 m to 2 km. To evaluate the model resolution, the path coverage test and checkerboard resolution test were performed.

**Figure 5:**
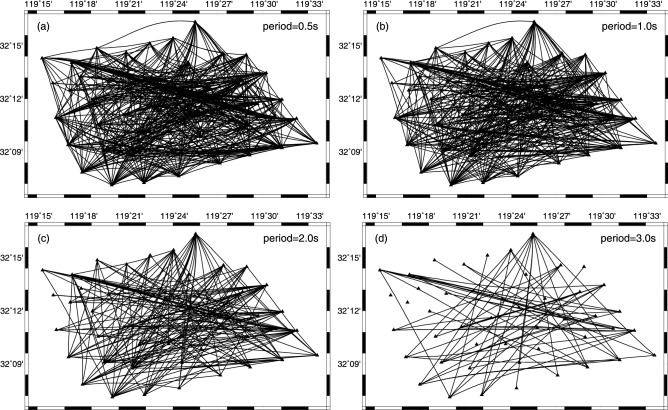
Ray-path coverage for four selected periods: (**a**) 0.5 s, (**b**) 1.0 s, (**c**) 2.0 s, and (**d**) 3.0 s. The triangles indicate stations, and the black lines represent ray paths.

**Figure 6:**
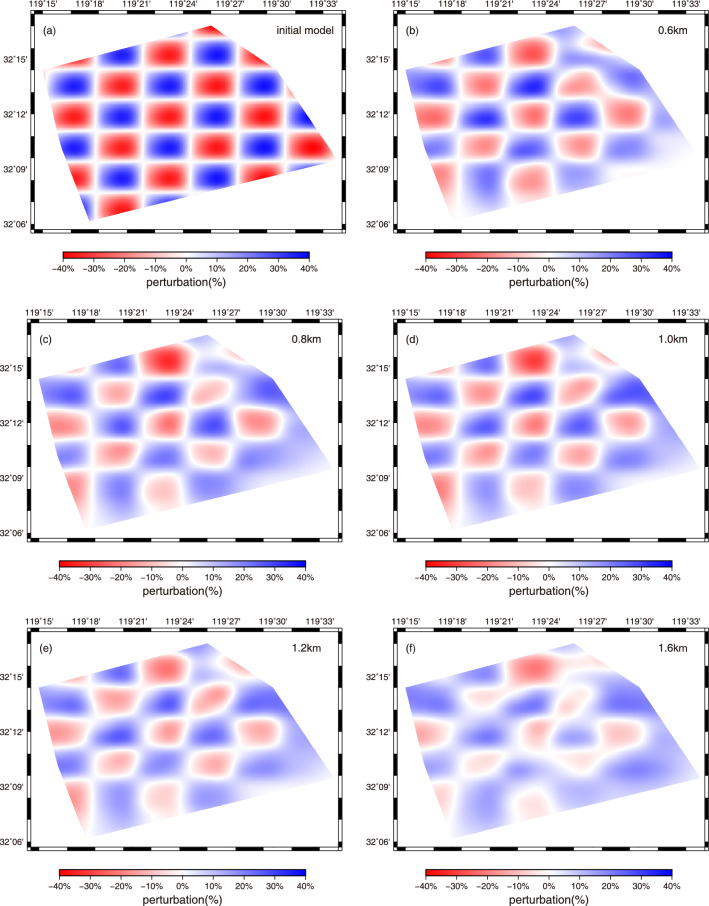
Checkerboard resolution tests of the inversion. a The initial checkerboard model and b-f the recovered checkerboard models at depths of 0.6, 0.8, 1.0, 1.2, and 1.6 km, respectively.

**Figure 7:**
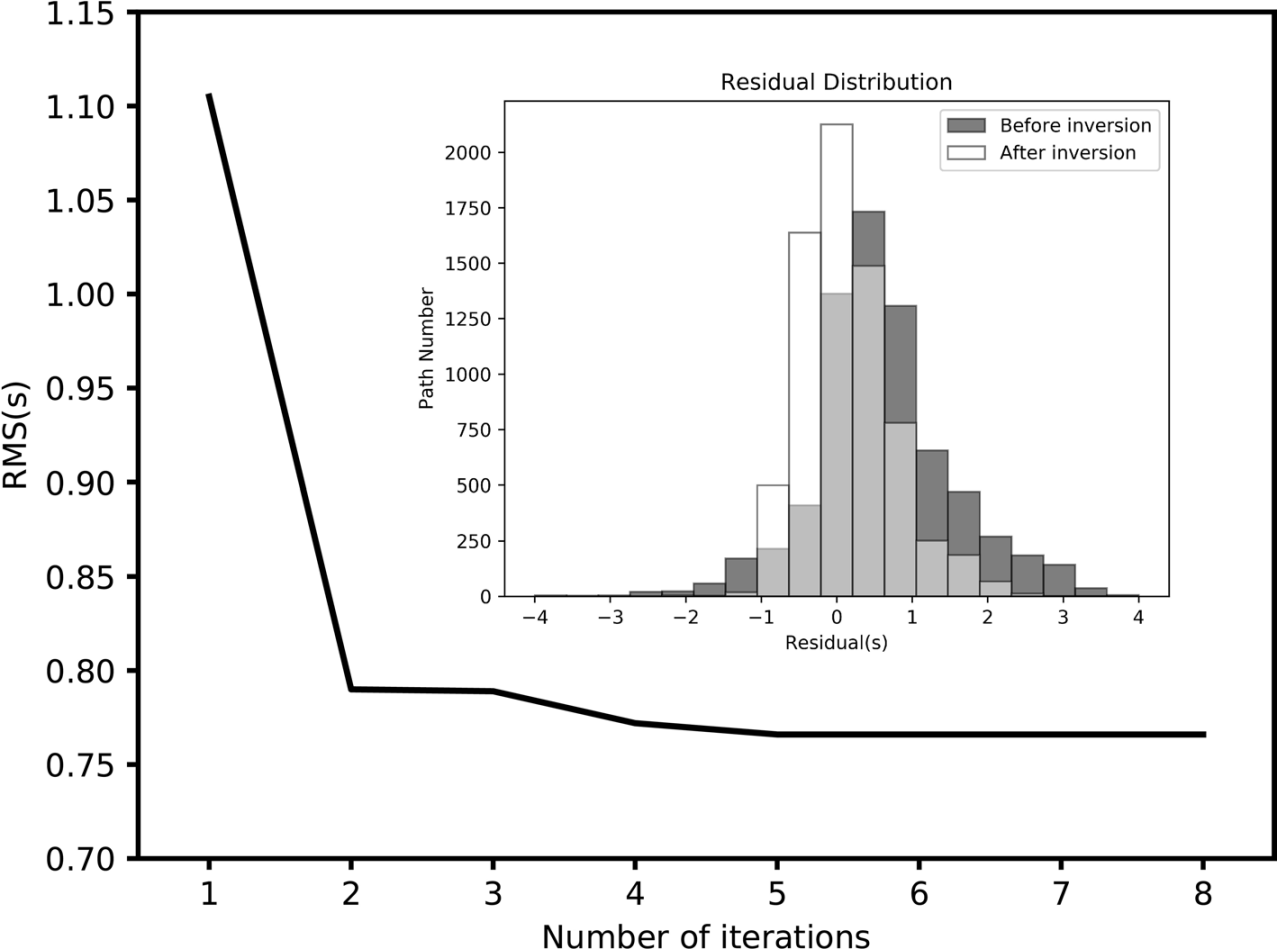
Variation of the RMS value of surface-wave travel-time residuals through the iterations. The inset shows the histograms of travel-time residuals before (in gray) and after inversion (in white).

Figure 5 presents the path coverage of group velocity measurements at four selected periods (0.5, 1.0, 2.0, 3.0) based on the final 3D velocity model. It can be clearly seen that the path number decreases with the increase of the period (from 484 at 0.5 s to 59 at 3.0 s). And generally speaking, the ray coverage at these periods is relatively good except for some marginal area, indicating that in the most of the study area, this dataset has the capability to resolve the structure. To further test the model resolution at different depths, the checkerboard resolution test was performed. The synthetic checkerboard model was set with − 40% and + 40% anomalies and each anomaly has a size of 3 km and 5 km in the latitude and at longitude direction, respectively. Figure 6 shows the initial checkerboard model and its recovery after inversion at five different depths. We can notice that, the resolution of checkerboard models decreases as the inversion depth increases and for each inversion depth, checkerboard models are recovered better in the central part of the study area compared with the marginal area, which is closely related to the path coverage. The results of path coverage test and checkerboard test suggest that, in the depth range of 0.6–1.6 km, the structures of most regions can be resolved well, while that of some marginal area is not resolved so well (less well fitted) due to insufficient ray paths. And then we inverted the real group-velocity dispersion data using the direct surface-wave tomography method mentioned above. After eight iterations, the root-mean-square RMS residual value decreases from about 1.1 s to about 0.75 s and remains stable, indicating that the inversion converges to a stable condition. And compared with that before iterations, the residual distribution after iterations is obviously closer to zero and follows the Gaussian distribution, which also means our inversion process has good convergence and the data fitting is relatively good (Fig. 7).

## Results and discussion

**Figure 8:**
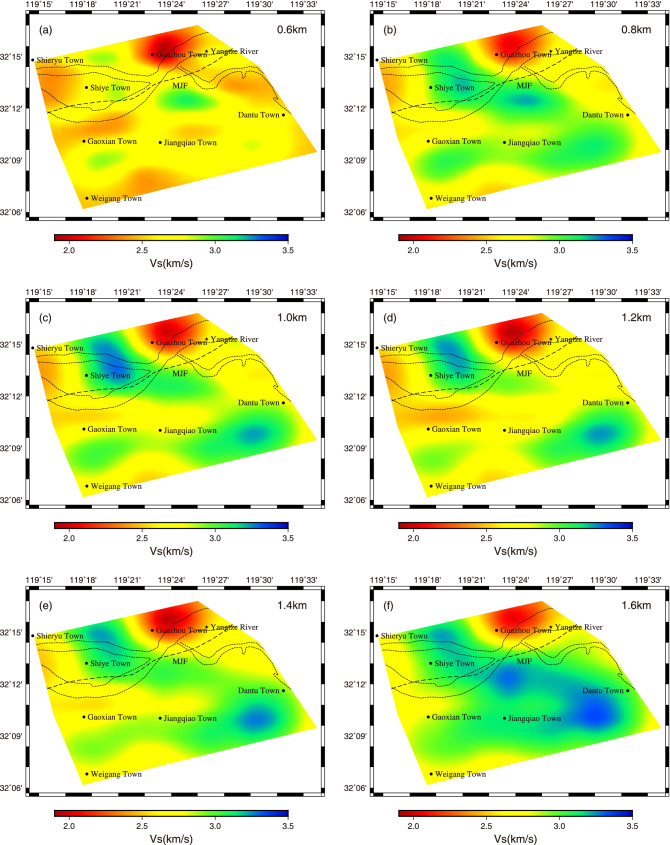
Map view of the ${V}_{s}$ model at depth of (**a**) 0.6, (**b**) 0.8, (**c**) 1.0, (**d**) 1.2, (**e**) 1.4, and (**f**) 1.6 km.

Figure 8 shows the V_s_ model at different depths in the study area. It can be seen that (1) The crust medium in the study area presents strong lateral velocity heterogeneity. Taking MJF as the boundary, the V_s_ on the north side is distributed in the near north–south direction with relatively low velocity, which may be related to the Yizheng depression, while on the south side, it is distributed in the near east–west direction with relatively high velocity, which could be related to the NMR; (2) Starting from the depth of 1.0 km, two high-velocity anomalies with a velocity of about 3.3 km/s began to appear in the northwest and southeast of the study area, and extended to the center forming a whole at a depth of 1.6 km. Since Mesozoic, the Lower Yangtze fault block has developed strong intermediate-acid magmatic activity and the MJF has invaded a lot of magmatic rocks near Shiye Town^22^. We think that the high-velocity anomaly in the northwest may be related to the magmatic rocks intruded in this area. While the high-velocity anomaly in the southeast probably be relevant to Silurian sandstone (S_1_g, S_2f._) and Devonian shale (D_3_w) which widely developed in this area. And this lithologic distribution possibly be related to the intersection of DJF and RSF in this area (Fig. 1); (3) The MJF is developed in the V_s_ anomaly gradient zone. Specifically, the west section of MJF is located on the transition zone between two relatively low-velocity areas on the north and south sides; the middle section is located on the transition zone between the relatively high-velocity area on the north side and the relatively low-velocity area on the south side; and the east section is located on the edge of the low-velocity anomaly on the north side.

**Figure 9:**
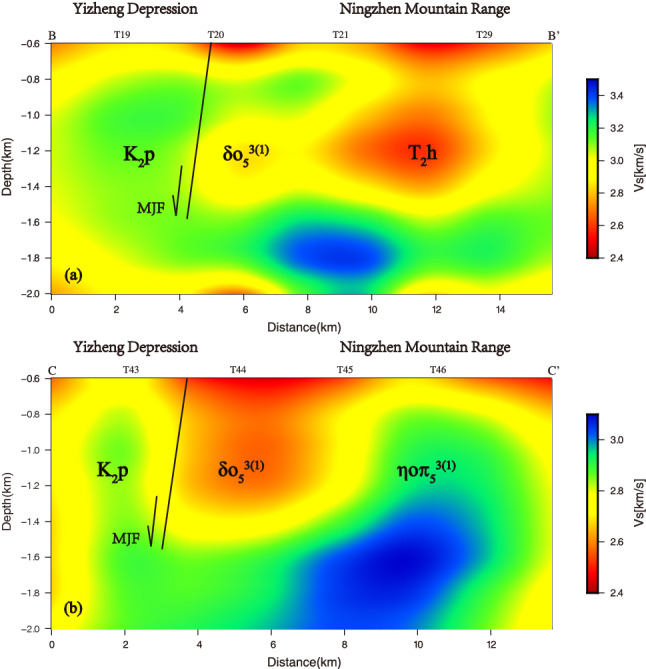
Cross-section of the ${V}_{s}$ model along (**a**) BB’ and (**b**) CC’. The MJF, K_2_p, δo_5_^3 (1)^, T_2_h, and ηοπ_5_^3(1)^ represent the Mufushan-jiaoshan fault, the Pukou Formation sandstone of Mesozoic, the quartz diorite of late Yanshan, the Huangmaqing formation sandstone of Middle Triassic, and the porphyritic quartz diorite, respectively.

**Figure 10:**
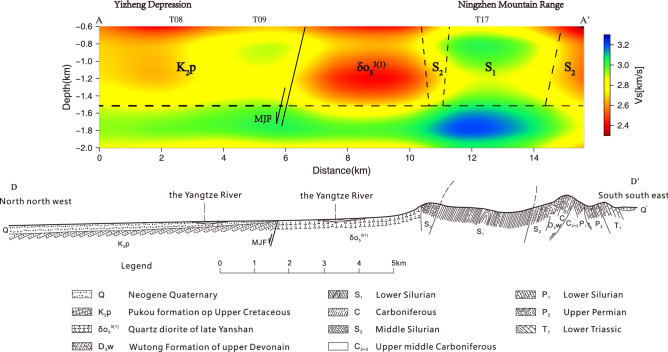
Cross-section of the ${V}_{s}$ model along AA’ and the local geological profile DD’^36^_._

In order to study the relationship between MJF and V_s_ in depth direction, we drew three cross-sections of V_s_ model along AA' (15 km), BB' (15 km), and CC' (13.5 km) along NW–SE direction (Fig. 9), and compared the AA' section with bedrock geological section DD' (Fig. 10). The profile DD' is located about 2 km east of AA' section, with a total length of 18.9 km, and the direction is basically the same as that of section AA'. See Fig. 1 for the location of profiles AA', BB', CC', and DD'.

We can obviously see from Fig. 10 that in the depth range of 0.6–1.5 km, the V_s_ model along profile AA' has a good correspondence with bedrock geological profile DD'. According to the distribution of velocity anomaly along profile AA', we divide it into five parts with dotted lines, which correspond to the Pukou Formation sandstone of Mesozoic (K_2_p), quartz diorite of late Yanshan (δo_5_^3 (1)^), sandstone of middle Silurian (S_2_), sandstone of lower Silurian (S_1_), and sandstone of middle Silurian (S_2_) in bedrock geological profile DD' from north to south, respectively. In addition, it is worth noting that the tendency of velocity interface in profile AA' is also consistent with that of lithology interface in profile DD' (such as the interface between K_2_p and δo_5_^3(1)^, δo_5_^3 (1)^ and S_2_, S_1_ and S_2_). The good correspondence between profiles AA' and DD' shows that the V_s_ model can reflect the change of regional geology to a certain extent. And the V_s_ models along profiles BB' and CC' are similar to those of profile AA', and also have a good corresponding relationship with bedrock lithology. Taking the MJF as the boundary: on the north side of profile BB' is the Pukou Formation sandstone of Mesozoic (K_2_p), showing a relatively high velocity, on the south side is the quartz diorite of late Yanshan (δo_5_^3 (1)^) and the Huangmaqing formation sandstone of Middle Triassic (T_2_h), both exhibiting relatively low velocity, and the latter is slower than the former; on the north side of CC' section is the Pukou Formation sandstone of Mesozoic (K_2_p), showing a relatively high velocity, and on the south side is the quartz diorite of late Yanshan (δo_5_^3 (1)^) showing a relatively low velocity and porphyritic quartz diorite (ηοπ_5_^3(1)^) showing a relatively high velocity. While in the depth range of 1.5–2.0 km, the velocity distribution characteristics of the three sections are similar, showing relatively low velocity in the north and relatively high velocity in the south. According to the characteristics of V_s_ distribution, it can be determined that the lower regions under station T09 (section AA’), T20 (section BB’), and the area between T43 and T44 (section CC’) are the development zones of MJF.

In general, the V_s_ model in study area is obviously non-uniform in both horizontal and vertical directions. In the depth range of 0.6–1.5 km, the medium on the north side of MJF fault presents relatively high velocity, while on the south side, it presents a pattern of alternating low and high velocity distribution, which is consistent with the multi-stage geological tectonic activities during the formation of MJF fault and NMR; in the depth range of 1.5–2.0 km, the velocity structure is relatively simple, with a relatively low velocity in the north and a relatively high velocity in the south, indicating that the lithology tends to be unified with the increase of depth, which is consistent with the structural background of Yizheng depression in the north of the fault and NMR in the south.

## Conclusion

In this study, we used group-velocity ambient noise tomography to investigate the structure of MJF in Zhenjiang area, China. 510 group velocity dispersion curves in the period band 0.5–5 s were used to invert 3D V_s_ structure by the direct surface-wave tomographic method. Our results reveal that, in the study area, the distribution of the V_s_ in different depth regions has different characteristics, which is consistent with the geological background of the area. And there are obvious differences in V_s_ distribution between the north and south sides of MJF. According to the three cross-sections of V_s_ model, the location of the development zone of MJF is also determined. The good agreement between the V_s_ model and the distribution of MJF as well as the geological background in the study indicate that, the ambient noise tomography method can be used as an effective method for detecting hidden faults in urban environments.

## References

[1] Zhao J (2020). Coupling fraction and relocking process of the Longmenshan Fault Zone following the 2008 Mw7.9 Wenchuan earthquake. J. Geodyn..

[2] ten Brink U, Wei Y, Fan W, Granja-Bruna J-L, Miller N (2020). Mysterious tsunami in the Caribbean Sea following the 2010 Haiti earthquake possibly generated by dynamically triggered early aftershocks. Earth Planet. Sci. Lett..

[3] Jiang G, Xu S, Jin Y, Chen D, Lu J (2016). Electric field response characteristics of buried fault with thin overburden layer—the example of Feihuanghe fault. Progr. Geophys..

[4] Weaver RL, Lobkis OI (2001). Ultrasonics without a source: Thermal fluctuation correlations at mhz frequencies. Phys. Rev. Lett..

[5] Rickett J, Claerbout JF (1999). Acoustic daylight imaging via spectral factorization; helioseismology and reservoir monitoring. Geophysics.

[6] Fichtner A, Stehly L, Ermert L, Boehm C (2017). Generalized interferometry—I: Theory for interstation correlations. Geophys. J. Int..

[7] Shapiro NM, Campillo M, Stehly L, Ritzwoller MH (2005). High-resolution surface-wave tomography from ambient seismic noise. Science.

[8] Sabra KG, Gerstoft P, Roux P, Kuperman WA, Fehler MC (2005). Surface wave tomography from microseisms in Southern California. Geophys. Res. Lett..

[9] Yao H, van der Hilst RD, de Hoop MV (2006). Surface-wave array tomography in SE Tibet from ambient seismic noise and two-station analysis—I Phase velocity maps. Geophys. J. Int..

[10] Bensen GD, Ritzwoller MH, Shapiro NM (2008). Broadband ambient noise surface wave tomography across the United States. J. Geophys. Res. Solid Earth.

[11] Moschetti MP, Ritzwoller MH, Shapiro NM (2007). Surface wave tomography of the western United States from ambient seismic noise: Rayleigh wave group velocity maps. Geochem. Geophys. Geosyst..

[12] Yao H, Beghein C, Van Der Hilst RD (2008). Surface wave array tomography in SE Tibet from ambient seismic noise and two-station analysis—II. Crustal and upper-mantle structure. Geophys. J. Int..

[13] Gu N (2018). Shallow crustal structure of the Tanlu Fault Zone Near Chao lake in Eastern China by direct surface wave tomography from local dense array ambient noise analysis. Pure Appl. Geophys..

[14] Huang YC (2010). Phase velocity variation at periods of 0.5–3 s in the Taipei Basin of Taiwan from correlation of ambient seismic noise. Bull. Seismol. Soc. Am..

[15] Li C (2016). 3D near-surface shear-wave velocity structure from ambient-noise tomography and Borehole data in the Hefei Urban Area China. Seismol. Res. Lett..

[16] Liu Y, Zhang H, Fang H, Yao H, Gao J (2018). Ambient noise tomography of three-dimensional near-surface shear-wave velocity structure around the hydraulic fracturing site using surface microseismic monitoring array. J. Appl. Geophys..

[17] Lin F-C, Moschetti MP, Ritzwoller MH (2008). Surface wave tomography of the western United States from ambient seismic noise: Rayleigh and Love wave phase velocity maps. Geophys. J. Int..

[18] Lin F-C, Li D, Clayton RW, Hollis D (2013). High-resolution 3D shallow crustal structure in Long Beach, California: Application of ambient noise tomography on a dense seismic array. Geophysics.

[19] Nakata N, Chang JP, Lawrence JF, Boué P (2015). Body wave extraction and tomography at Long Beach, California, with ambient-noise interferometry. J. Geophys. Res. Solid Earth.

[20] Mordret A, Landès M, Shapiro NM, Singh SC, Roux P (2014). Ambient noise surface wave tomography to determine the shallow shear velocity structure at Valhall: Depth inversion with a neighbourhood algorithm. Geophys. J. Int..

[21] Chmiel M (2019). Ambient noise multimode Rayleigh and Love wave tomography to determine the shear velocity structure above the Groningen gas field. Geophys. J. Int..

[22] Zong K, Zong W, Kang C, Bai S (2016). Research on the major active faults in Zhenjiang, Jiangsu and their late quaternary activities. J. Geomech..

[23] Huang, Y. & Wang, Y. Analysis of crustal movement and geological development history in Ningzhen area. *Journal of Shengli Oilfield staff University* (in Chinese) (2001).

[24] Miao Q (2016). Neotectonic movement characteristics of the Dalu-Yaoqiao Fault in eastern Zhenjiang Jiangsu Province. J. Geol..

[25] Bensen GD (2007). Processing seismic ambient noise data to obtain reliable broad-band surface wave dispersion measurements. Geophys. J. Int..

[26] Zhang Y (2018). 3-D crustal shear-wave velocity structure of the Taiwan Strait and Fujian, SE China, revealed by ambient noise tomography. J. Geophys. Res. Solid Earth.

[27] Zeng Q, Chu R, Cheng M, Wei Z (2020). Seismic ambient noise tomography for shallow velocity stuctures beneath Weiyuan Sichuan. Chin. J. Geophys..

[28] Frank SD, Foster AE, Ferris AN, Johnson M (2009). Frequency-dependent asymmetry of seismic cross-correlation functions associated with noise directionality. Bull. Seismol. Soc. Am..

[29] Picozzi M, Parolai S, Bindi D, Strollo A (2009). Characterization of shallow geology by high-frequency seismic noise tomography. Geophys. J. Int..

[30] Yao H, Van Der Hilst RD (2009). Analysis of ambient noise energy distribution and phase velocity bias in ambient noise tomography, with application to SE Tibet. Geophys. J. Int..

[31] Yao H, Gouédard P, Collins JA, McGuire JJ, van der Hilst RD (2011). Structure of young East Pacific Rise lithosphere from ambient noise correlation analysis of fundamental- and higher-mode Scholte-Rayleigh waves. C.R. Geosci..

[32] Dziewonski A, Bloch S, Landisman M (1969). A technique for the analysis of transient seismic signals. Bull. Seismol. Soc. Am..

[33] Fang H, Yao H, Zhang H, Huang Y-C, van der Hilst RD (2015). Direct inversion of surface wave dispersion for three-dimensional shallow crustal structure based on ray tracing: methodology and application. Geophys. J. Int..

[34] Rawlinson N, Sambridge M (2004). Wave front evolution in strongly heterogeneous layered media using the fast marching method. Geophys. J. Int..

[35] Brocher TM (2005). Empirical relations between elastic wavespeeds and density in the Earth's crust. Bull. Seismol. Soc. Am..

[36] Jiangsu Provincial Bureau of Geology and mineral resources. Memoir on geology of Nanjing-Zhenjiang mountains (in Chinese) (Phoenix Science Press, 1992).

